# (Sulfasalazinato-κ*O*)bis­(triphenyl­phosphine-κ*P*)copper(I)

**DOI:** 10.1107/S1600536810013899

**Published:** 2010-04-21

**Authors:** Ke-Bin Huang, Yan-Shu Tan, Xiao-Yan Song, Yan-Cheng Liu, Zhen-Feng Chen

**Affiliations:** a Key Laboratory for the Chemistry and Molecular Engineering of Medicinal Resources (Ministry of Education of China), School of Chemistry & Chemical Engineering, Guangxi Normal University, Guilin 541004, People’s Republic of China

## Abstract

The title mixed-ligand copper(I) complex, [Cu(C_18_H_13_N_4_O_5_S)(C_18_H_15_P)_2_], was synthesized *via* solvothermal reaction of [Cu(PPh_3_)_2_(MeCN)_2_]ClO_4_ and sulfasalazine [systematic name: 2-hydr­oxy-5-(2-{4-[(2-pyridylamino)sulfon­yl]phen­yl}diazen­yl)benzoic acid]. The mononuclear complex displays a trigonal coordination geometry for the Cu(I) atom, which is surrounded by two P-atom donors from two different PPh_3_ ligands and one O-atom donor from the monodentate carboxyl­ate group of the sulfasalazinate ligand. The latter ligand is found in a zwitterionic form, with a deprotonated amine N atom and a protonated pyridine N atom. Such a feature was previously described for free sulfasalazine. The crystal structure is stabilized by C—H⋯O, C—H⋯N, N—H⋯N and O—H⋯O hydrogen bonds.

## Related literature

For applications of sulfasalazine, see: Neva *et al.* (2000 ▶); Mansfield *et al.* (2002 ▶). For crystal structures of metal complexes with sulfasalazine, see: Chen *et al.* (2003 ▶, 2008 ▶); Kang *et al.* (2006 ▶, 2008*a* ▶,*b* ▶); Wang *et al.* (2005 ▶); Yuan *et al.* (2006 ▶). For the crystal structure of free sulfasalazine, see: van der Sluis & Spek (1990 ▶). For the structure of a zwitterion related to sulfasalazine, see: Eliopoulos *et al.* (1983 ▶). For spectroscopic evidences supporting the presence of a proton­ated pyridine, see: Franklin & Richardson (1980 ▶). 
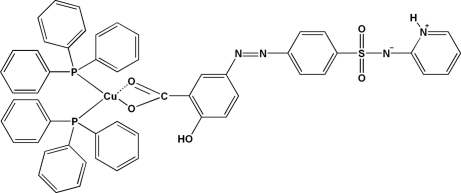


## Experimental

### 

#### Crystal data

[Cu(C_18_H_13_N_4_O_5_S)(C_18_H_15_P)_2_]
*M* *_r_* = 985.46
Triclinic, 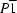

*a* = 14.0126 (5) Å
*b* = 14.2236 (12) Å
*c* = 14.2302 (4) Å
α = 81.068 (11)°
β = 61.606 (7)°
γ = 75.011 (10)°
*V* = 2408.4 (2) Å^3^
*Z* = 2
Mo *K*α radiation
μ = 0.62 mm^−1^
*T* = 193 K
0.40 × 0.30 × 0.10 mm

#### Data collection

Rigaku Mercury CCD diffractometer
Absorption correction: multi-scan (*CrystalClear*; Rigaku, 2000 ▶) *T*_min_ = 0.791, *T*_max_ = 0.941
24096 measured reflections
8763 independent reflections
6866 reflections with *I* > 2σ(*I*)
*R*_int_ = 0.044

#### Refinement

*R*[*F* ^2^ > 2σ(*F* ^2^)] = 0.054
*wR*(*F* ^2^) = 0.109
*S* = 1.10
8763 reflections
610 parameters
H atoms treated by a mixture of independent and constrained refinement
Δρ_max_ = 0.31 e Å^−3^
Δρ_min_ = −0.33 e Å^−3^

### 

Data collection: *CrystalClear* (Rigaku, 2000 ▶); cell refinement: *CrystalClear*; data reduction: *CrystalClear*; program(s) used to solve structure: *SHELXS97* (Sheldrick, 2008 ▶); program(s) used to refine structure: *SHELXL97* (Sheldrick, 2008 ▶); molecular graphics: *SHELXTL* (Sheldrick, 2008 ▶); software used to prepare material for publication: *SHELXTL*.

## Supplementary Material

Crystal structure: contains datablocks I, global. DOI: 10.1107/S1600536810013899/bh2279sup1.cif

Structure factors: contains datablocks I. DOI: 10.1107/S1600536810013899/bh2279Isup2.hkl

Additional supplementary materials:  crystallographic information; 3D view; checkCIF report

## Figures and Tables

**Table 1:** Hydrogen-bond geometry (Å, °)

*D*—H⋯*A*	*D*—H	H⋯*A*	*D*⋯*A*	*D*—H⋯*A*
C46—H46⋯N3^i^	0.95	2.59	3.542 (5)	178
C5—H5⋯O5^ii^	0.95	2.49	3.429 (4)	169
N4—H1⋯N3^iii^	0.91 (4)	2.20 (4)	3.112 (4)	171 (3)
O3—H3⋯O1	0.84	1.76	2.510 (3)	147

Symmetry codes: (i) 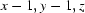; (ii) 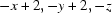; (iii) 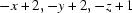.

**Fig. 1.**
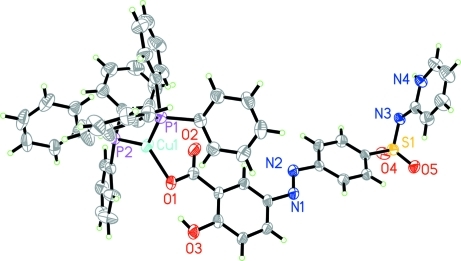
The molecular structure of the title complex, showing the atom-labelling scheme. Displacement ellipsoids are drawn at the 50% probability level.

**Fig. 2.**
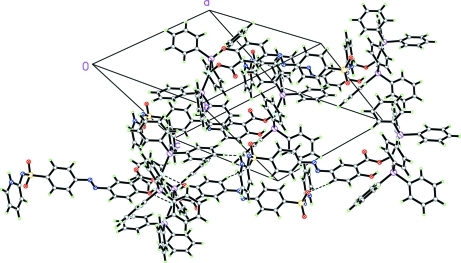
A packing diagram of the title complex. Some Hydrogen bonds are shown as dashed lines.

## supplementary crystallographic information

### Comment

Sulfasalazine (systematic name: 2-hydroxy-5-[2-[4-[(2-pyridinylamino)sulfonyl]phenyl]diazenyl]-benzoic acid) is a drug used in treatment of joint diseases such as rheumatoid arthritis and spondyloarthropathies (Neva *et al.*, 2000), as well as in inflammatory bowel diseases (Mansfield *et al.*, 2002). Several crystal structures of metal complexes with sulfasalazine have been reported, with the sulfasalazine exhibiting diversity in its coordination behavior, giving rise to the formation of both monomeric and polymeric structures (Chen *et al.*, 2003, 2008; Kang *et al.*, 2006, 2008*a*,*b*; Wang *et al.*, 2005; Yuan *et al.*, 2006).

The copper ion in the title complex displays a trigonal geometry, being linked to two P atoms of two PPh_3_ ligands, and one O atom of the sulfasalazine ligand (Fig. 1), Due to the great steric effect of the bulky PPh_3_ ligands, there is a weak bond Cu1—O2 [2.624 (2)Å]. The geometric parameters of sulfasalazine are as expected, and comparable to the reported values (Chen *et al.*, 2008). They also compare well with those of free sulfasalazine (van der Sluis & Spek, 1990) and ethyl-3-[4,5]-dimethoxy-2-(4-methyl-2-pyridylsulphamoyl)phenyl]propionate (Eliopoulos *et al.*, 1983). The sulfasalazine ligand displays a zwitterionic form, with a deprotonated N atom (N3) and a protonated N atom (N4), which is further supported by a strong peak at 1525 cm^-1^ in the infrared spectrum of the complex (Franklin & Richardson 1980). The crystal structure is stabilized by C—H···O, C—H···N, N—H···N, and O—H···O hydrogen bonds (Table 1 and Fig. 2).

### Experimental

Samples of [Cu(PPh_3_)_2_(MeCN)_2_]ClO_4_ (0.1 mmol) and sulfasalazine (0.1 mmol) were placed in a thick-walled Pyrex tube (*ca* 20 cm long). After addition of CHCl_3_ (1 ml), the tube was frozen with liquid nitrogen, evacuated under vacuum and sealed with a torch. The tube was heated at 353 K for 2 days and then was slowly cooled down to room temperature. Orange-red block crystals were obtained. Yield: 45%. IR (KBr, cm^-1^): 3444(m), 3050(m), 1627(s), 1586(s), 1525(m), 1463(m), 1434(m), 1362(m), 1291(m), 1269(m), 1198(w), 1169(m), 1135(s), 1083(m), 1002(m), 963(m), 930(w), 848(m), 805(m), 772(m), 746(s), 695(s), 645(w), 610(s), 582(m), 565(m), 510(m).

### Refinement

H atoms bonded to C atoms were positioned geometrically and refined using a riding model with *U*_iso_(H) = 1.2*U*_eq_(C) (C—H = 0.95 Å). The hydroxyl H atom H3 was placed in calculated position, with O—H distance of 0.84 Å, and *U*_iso_(H3) = 1.5*U*_eq_(O3). H atom attached to N4 in the pyridine ring (H1) was located in a difference map and refined freely.

### Crystal data

**Table:**

[Cu(C_18_H_13_N_4_O_5_S)(C_18_H_15_P)_2_]	*Z* = 2
*M**_r_* = 985.46	*F*(000) = 1020
Triclinic, *P*1	*D*_x_ = 1.359 Mg m^−^^3^
Hall symbol: -P 1	Mo *K*α radiation, λ = 0.71070 Å
*a* = 14.0126 (5) Å	Cell parameters from 8593 reflections
*b* = 14.2236 (12) Å	θ = 3.0–25.3°
*c* = 14.2302 (4) Å	µ = 0.62 mm^−^^1^
α = 81.068 (11)°	*T* = 193 K
β = 61.606 (7)°	Block, orange-red
γ = 75.011 (10)°	0.40 × 0.30 × 0.10 mm
*V* = 2408.4 (2) Å^3^	

### Data collection

**Table:**

Rigaku Mercury CCD diffractometer	8763 independent reflections
Radiation source: fine-focus sealed tube	6866 reflections with *I* > 2σ(*I*)
graphite	*R*_int_ = 0.044
Detector resolution: 7.31 pixels mm^-1^	θ_max_ = 25.4°, θ_min_ = 3.0°
ω scans	*h* = −15→16
Absorption correction: multi-scan (*CrystalClear*; Rigaku, 2000)	*k* = −16→17
*T*_min_ = 0.791, *T*_max_ = 0.941	*l* = −17→17
24096 measured reflections	

### Refinement

**Table:**

Refinement on *F*^2^	Primary atom site location: structure-invariant direct methods
Least-squares matrix: full	Secondary atom site location: difference Fourier map
*R*[*F*^2^ > 2σ(*F*^2^)] = 0.054	Hydrogen site location: inferred from neighbouring sites
*wR*(*F*^2^) = 0.109	H atoms treated by a mixture of independent and constrained refinement
*S* = 1.10	*w* = 1/[σ^2^(*F*_o_^2^) + (0.0333*P*)^2^ + 1.5872*P*] where *P* = (*F*_o_^2^ + 2*F*_c_^2^)/3
8763 reflections	(Δ/σ)_max_ < 0.001
610 parameters	Δρ_max_ = 0.31 e Å^−^^3^
0 restraints	Δρ_min_ = −0.33 e Å^−^^3^
0 constraints	

### Fractional atomic coordinates and isotropic or equivalent isotropic displacement parameters (Å^2^)

**Table:**

	*x*	*y*	*z*	*U*_iso_*/*U*_eq_	
Cu1	0.45981 (3)	0.41881 (3)	0.23424 (3)	0.03507 (12)	
S1	0.98672 (7)	1.04661 (6)	0.28783 (6)	0.0347 (2)	
P1	0.59805 (6)	0.29238 (6)	0.21974 (6)	0.0316 (2)	
P2	0.28157 (6)	0.44685 (6)	0.35221 (6)	0.02895 (19)	
O1	0.50582 (17)	0.52994 (15)	0.12432 (17)	0.0374 (5)	
O2	0.52453 (19)	0.56619 (17)	0.25992 (18)	0.0449 (6)	
O3	0.58620 (19)	0.61550 (17)	−0.05550 (17)	0.0419 (6)	
H3	0.5513	0.5767	−0.0085	0.063*	
O4	0.89660 (19)	1.12476 (16)	0.34663 (18)	0.0444 (6)	
O5	1.07566 (18)	1.07320 (17)	0.18968 (17)	0.0425 (6)	
N1	0.7910 (2)	0.80601 (18)	0.0838 (2)	0.0321 (6)	
N2	0.7683 (2)	0.80115 (19)	0.1806 (2)	0.0346 (6)	
N3	1.0242 (2)	0.98829 (19)	0.3734 (2)	0.0344 (6)	
N4	1.1440 (2)	0.8879 (2)	0.4284 (2)	0.0379 (7)	
C1	0.5420 (2)	0.5779 (2)	0.1661 (3)	0.0332 (7)	
C2	0.6124 (2)	0.6470 (2)	0.0913 (2)	0.0284 (7)	
C3	0.6324 (2)	0.6610 (2)	−0.0162 (2)	0.0323 (7)	
C4	0.7023 (2)	0.7211 (2)	−0.0851 (2)	0.0350 (8)	
H4	0.7152	0.7306	−0.1574	0.042*	
C5	0.7531 (2)	0.7671 (2)	−0.0491 (2)	0.0341 (7)	
H5	0.8014	0.8080	−0.0970	0.041*	
C6	0.7343 (2)	0.7543 (2)	0.0566 (2)	0.0297 (7)	
C7	0.6634 (2)	0.6941 (2)	0.1270 (2)	0.0306 (7)	
H7	0.6501	0.6855	0.1994	0.037*	
C8	0.8276 (2)	0.8567 (2)	0.2012 (2)	0.0305 (7)	
C9	0.9189 (3)	0.8911 (2)	0.1218 (2)	0.0378 (8)	
H9	0.9476	0.8753	0.0495	0.045*	
C10	0.9677 (3)	0.9485 (2)	0.1486 (2)	0.0369 (8)	
H10	1.0292	0.9733	0.0943	0.044*	
C11	0.9274 (2)	0.9699 (2)	0.2542 (2)	0.0296 (7)	
C12	0.8391 (3)	0.9323 (2)	0.3337 (3)	0.0385 (8)	
H12	0.8125	0.9457	0.4063	0.046*	
C13	0.7897 (3)	0.8750 (2)	0.3071 (3)	0.0396 (8)	
H13	0.7299	0.8484	0.3617	0.047*	
C14	1.1221 (3)	0.9230 (2)	0.3448 (3)	0.0357 (8)	
C15	1.2032 (3)	0.8868 (3)	0.2444 (3)	0.0481 (9)	
H15	1.1942	0.9106	0.1823	0.058*	
C16	1.2944 (3)	0.8177 (3)	0.2358 (3)	0.0588 (11)	
H16	1.3479	0.7935	0.1675	0.071*	
C17	1.3108 (3)	0.7820 (3)	0.3245 (3)	0.0590 (11)	
H17	1.3741	0.7331	0.3179	0.071*	
C18	1.2341 (3)	0.8186 (3)	0.4208 (3)	0.0516 (10)	
H18	1.2434	0.7958	0.4829	0.062*	
C19	0.7369 (2)	0.3189 (2)	0.1587 (2)	0.0342 (7)	
C20	0.8185 (3)	0.2702 (3)	0.1884 (3)	0.0436 (8)	
H20	0.8027	0.2221	0.2449	0.052*	
C21	0.9230 (3)	0.2918 (3)	0.1358 (3)	0.0513 (10)	
H21	0.9791	0.2576	0.1554	0.062*	
C22	0.9461 (3)	0.3628 (3)	0.0550 (3)	0.0499 (9)	
H22	1.0180	0.3772	0.0189	0.060*	
C23	0.8649 (3)	0.4125 (3)	0.0266 (3)	0.0484 (9)	
H23	0.8806	0.4618	−0.0287	0.058*	
C24	0.7604 (3)	0.3910 (2)	0.0784 (3)	0.0391 (8)	
H24	0.7045	0.4260	0.0588	0.047*	
C25	0.6144 (2)	0.1902 (2)	0.1472 (2)	0.0333 (7)	
C26	0.5200 (3)	0.1771 (3)	0.1471 (3)	0.0573 (11)	
H26	0.4521	0.2230	0.1802	0.069*	
C27	0.5235 (4)	0.0976 (3)	0.0991 (4)	0.0774 (15)	
H27	0.4578	0.0885	0.1008	0.093*	
C28	0.6225 (3)	0.0317 (3)	0.0489 (3)	0.0606 (11)	
H28	0.6252	−0.0230	0.0163	0.073*	
C29	0.7160 (3)	0.0456 (3)	0.0462 (3)	0.0461 (9)	
H29	0.7844	0.0009	0.0103	0.055*	
C30	0.7131 (3)	0.1236 (2)	0.0949 (3)	0.0415 (8)	
H30	0.7793	0.1318	0.0925	0.050*	
C31	0.5741 (2)	0.2396 (2)	0.3525 (2)	0.0354 (8)	
C32	0.5958 (3)	0.1413 (3)	0.3768 (3)	0.0493 (9)	
H32	0.6305	0.0960	0.3214	0.059*	
C33	0.5671 (3)	0.1079 (3)	0.4819 (3)	0.0630 (11)	
H33	0.5821	0.0400	0.4981	0.076*	
C34	0.5173 (3)	0.1727 (4)	0.5624 (3)	0.0656 (12)	
H34	0.4972	0.1495	0.6342	0.079*	
C35	0.4962 (3)	0.2710 (4)	0.5395 (3)	0.0643 (12)	
H35	0.4634	0.3160	0.5949	0.077*	
C36	0.5230 (3)	0.3039 (3)	0.4354 (3)	0.0487 (9)	
H36	0.5064	0.3718	0.4200	0.058*	
C37	0.2514 (2)	0.4620 (2)	0.4891 (2)	0.0325 (7)	
C38	0.3070 (3)	0.5206 (3)	0.5054 (3)	0.0524 (10)	
H38	0.3588	0.5518	0.4461	0.063*	
C39	0.2872 (4)	0.5338 (3)	0.6076 (3)	0.0711 (13)	
H39	0.3248	0.5748	0.6181	0.085*	
C40	0.2139 (4)	0.4884 (3)	0.6943 (3)	0.0626 (11)	
H40	0.2015	0.4972	0.7644	0.075*	
C41	0.1589 (3)	0.4301 (3)	0.6788 (3)	0.0601 (11)	
H41	0.1080	0.3985	0.7383	0.072*	
C42	0.1772 (3)	0.4175 (3)	0.5771 (3)	0.0481 (9)	
H42	0.1381	0.3774	0.5674	0.058*	
C43	0.2143 (2)	0.3488 (2)	0.3632 (2)	0.0335 (7)	
C44	0.2645 (3)	0.2546 (2)	0.3810 (3)	0.0430 (8)	
H44	0.3280	0.2443	0.3924	0.052*	
C45	0.2232 (4)	0.1755 (3)	0.3823 (3)	0.0581 (11)	
H45	0.2579	0.1114	0.3948	0.070*	
C46	0.1319 (4)	0.1906 (4)	0.3653 (3)	0.0652 (13)	
H46	0.1038	0.1365	0.3655	0.078*	
C47	0.0804 (3)	0.2830 (4)	0.3478 (3)	0.0592 (12)	
H47	0.0167	0.2926	0.3367	0.071*	
C48	0.1219 (3)	0.3628 (3)	0.3463 (3)	0.0449 (9)	
H48	0.0868	0.4268	0.3338	0.054*	
C49	0.1983 (2)	0.5553 (2)	0.3211 (2)	0.0330 (7)	
C50	0.2246 (3)	0.5793 (3)	0.2144 (3)	0.0470 (9)	
H50	0.2866	0.5408	0.1599	0.056*	
C51	0.1603 (3)	0.6595 (3)	0.1876 (3)	0.0589 (11)	
H51	0.1781	0.6753	0.1146	0.071*	
C52	0.0713 (3)	0.7161 (3)	0.2654 (3)	0.0510 (9)	
H52	0.0266	0.7700	0.2464	0.061*	
C53	0.0469 (3)	0.6953 (3)	0.3702 (3)	0.0462 (9)	
H53	−0.0134	0.7361	0.4237	0.055*	
C54	0.1094 (3)	0.6151 (2)	0.3990 (3)	0.0397 (8)	
H54	0.0916	0.6010	0.4722	0.048*	
H1	1.094 (3)	0.918 (3)	0.491 (3)	0.056 (11)*	

### Atomic displacement parameters (Å^2^)

**Table:**

	*U*^11^	*U*^22^	*U*^33^	*U*^12^	*U*^13^	*U*^23^
Cu1	0.0288 (2)	0.0294 (2)	0.0395 (2)	−0.00721 (16)	−0.01025 (17)	0.00251 (17)
S1	0.0439 (5)	0.0330 (5)	0.0374 (4)	−0.0145 (4)	−0.0237 (4)	−0.0004 (4)
P1	0.0293 (4)	0.0304 (5)	0.0331 (4)	−0.0045 (3)	−0.0130 (3)	−0.0025 (4)
P2	0.0279 (4)	0.0282 (4)	0.0317 (4)	−0.0079 (3)	−0.0129 (3)	−0.0027 (3)
O1	0.0399 (13)	0.0356 (13)	0.0462 (13)	−0.0202 (10)	−0.0229 (11)	0.0044 (10)
O2	0.0499 (14)	0.0508 (15)	0.0358 (13)	−0.0267 (12)	−0.0155 (11)	0.0071 (11)
O3	0.0508 (14)	0.0491 (15)	0.0378 (13)	−0.0297 (11)	−0.0201 (11)	−0.0023 (11)
O4	0.0574 (15)	0.0357 (13)	0.0475 (14)	−0.0035 (11)	−0.0314 (12)	−0.0068 (11)
O5	0.0503 (14)	0.0466 (14)	0.0415 (13)	−0.0276 (11)	−0.0231 (11)	0.0060 (11)
N1	0.0330 (14)	0.0349 (15)	0.0330 (15)	−0.0133 (11)	−0.0163 (12)	0.0007 (12)
N2	0.0392 (15)	0.0382 (16)	0.0343 (15)	−0.0168 (12)	−0.0196 (12)	0.0009 (12)
N3	0.0380 (15)	0.0386 (16)	0.0373 (15)	−0.0115 (12)	−0.0236 (12)	−0.0023 (12)
N4	0.0391 (16)	0.0434 (18)	0.0375 (16)	−0.0070 (13)	−0.0223 (14)	−0.0051 (14)
C1	0.0295 (17)	0.0315 (18)	0.0385 (19)	−0.0083 (13)	−0.0144 (14)	−0.0015 (15)
C2	0.0245 (15)	0.0286 (17)	0.0318 (16)	−0.0101 (12)	−0.0103 (13)	−0.0014 (13)
C3	0.0292 (16)	0.0333 (18)	0.0362 (17)	−0.0114 (13)	−0.0125 (14)	−0.0071 (14)
C4	0.0361 (18)	0.047 (2)	0.0249 (16)	−0.0219 (15)	−0.0100 (14)	−0.0003 (14)
C5	0.0332 (17)	0.042 (2)	0.0287 (17)	−0.0184 (14)	−0.0114 (14)	0.0030 (14)
C6	0.0311 (16)	0.0277 (17)	0.0365 (17)	−0.0111 (13)	−0.0173 (14)	−0.0032 (14)
C7	0.0321 (17)	0.0306 (17)	0.0297 (16)	−0.0104 (13)	−0.0123 (13)	−0.0020 (13)
C8	0.0328 (17)	0.0316 (18)	0.0356 (18)	−0.0123 (13)	−0.0208 (14)	0.0021 (14)
C9	0.0432 (19)	0.050 (2)	0.0280 (17)	−0.0198 (16)	−0.0181 (15)	0.0000 (15)
C10	0.0378 (18)	0.049 (2)	0.0329 (18)	−0.0209 (15)	−0.0192 (15)	0.0046 (15)
C11	0.0326 (17)	0.0302 (17)	0.0337 (17)	−0.0098 (13)	−0.0203 (14)	0.0006 (14)
C12	0.0395 (19)	0.048 (2)	0.0298 (17)	−0.0129 (16)	−0.0140 (15)	−0.0076 (15)
C13	0.0406 (19)	0.049 (2)	0.0352 (18)	−0.0226 (16)	−0.0170 (15)	0.0018 (16)
C14	0.0416 (19)	0.039 (2)	0.0388 (19)	−0.0188 (15)	−0.0224 (16)	−0.0017 (15)
C15	0.050 (2)	0.060 (3)	0.038 (2)	−0.0110 (19)	−0.0214 (17)	−0.0101 (18)
C16	0.047 (2)	0.073 (3)	0.050 (2)	0.001 (2)	−0.0186 (19)	−0.023 (2)
C17	0.050 (2)	0.065 (3)	0.066 (3)	0.0062 (19)	−0.033 (2)	−0.023 (2)
C18	0.051 (2)	0.056 (2)	0.054 (2)	−0.0009 (19)	−0.0325 (19)	−0.0096 (19)
C19	0.0291 (17)	0.0357 (19)	0.0352 (17)	−0.0036 (14)	−0.0123 (14)	−0.0079 (15)
C20	0.0372 (19)	0.040 (2)	0.055 (2)	−0.0057 (15)	−0.0233 (17)	0.0008 (17)
C21	0.034 (2)	0.055 (2)	0.068 (3)	−0.0056 (17)	−0.0276 (18)	−0.005 (2)
C22	0.0301 (19)	0.057 (3)	0.058 (2)	−0.0124 (17)	−0.0122 (17)	−0.010 (2)
C23	0.043 (2)	0.049 (2)	0.044 (2)	−0.0169 (17)	−0.0096 (17)	−0.0014 (17)
C24	0.0353 (19)	0.042 (2)	0.0374 (18)	−0.0070 (15)	−0.0150 (15)	−0.0028 (16)
C25	0.0344 (18)	0.0316 (18)	0.0309 (17)	−0.0025 (14)	−0.0143 (14)	−0.0027 (14)
C26	0.040 (2)	0.061 (3)	0.078 (3)	0.0046 (18)	−0.031 (2)	−0.031 (2)
C27	0.058 (3)	0.079 (3)	0.118 (4)	0.001 (2)	−0.053 (3)	−0.051 (3)
C28	0.066 (3)	0.049 (2)	0.074 (3)	0.003 (2)	−0.038 (2)	−0.026 (2)
C29	0.043 (2)	0.040 (2)	0.049 (2)	−0.0020 (16)	−0.0175 (17)	−0.0120 (17)
C30	0.0349 (18)	0.037 (2)	0.048 (2)	−0.0036 (15)	−0.0163 (16)	−0.0066 (16)
C31	0.0283 (17)	0.042 (2)	0.0374 (18)	−0.0084 (14)	−0.0158 (14)	−0.0012 (15)
C32	0.057 (2)	0.051 (2)	0.041 (2)	−0.0156 (18)	−0.0222 (18)	0.0023 (18)
C33	0.071 (3)	0.067 (3)	0.055 (3)	−0.029 (2)	−0.031 (2)	0.019 (2)
C34	0.052 (2)	0.107 (4)	0.039 (2)	−0.028 (2)	−0.0208 (19)	0.012 (3)
C35	0.055 (2)	0.099 (4)	0.039 (2)	−0.004 (2)	−0.0241 (19)	−0.015 (2)
C36	0.046 (2)	0.058 (2)	0.043 (2)	−0.0033 (18)	−0.0228 (17)	−0.0108 (18)
C37	0.0315 (17)	0.0336 (18)	0.0336 (17)	−0.0065 (14)	−0.0145 (14)	−0.0062 (14)
C38	0.056 (2)	0.060 (3)	0.046 (2)	−0.0306 (19)	−0.0154 (18)	−0.0124 (19)
C39	0.081 (3)	0.090 (3)	0.061 (3)	−0.036 (3)	−0.031 (2)	−0.027 (3)
C40	0.083 (3)	0.070 (3)	0.041 (2)	−0.012 (2)	−0.032 (2)	−0.015 (2)
C41	0.081 (3)	0.064 (3)	0.031 (2)	−0.027 (2)	−0.0165 (19)	−0.0024 (19)
C42	0.058 (2)	0.052 (2)	0.040 (2)	−0.0225 (18)	−0.0208 (18)	−0.0043 (17)
C43	0.0325 (17)	0.041 (2)	0.0241 (16)	−0.0152 (14)	−0.0051 (13)	−0.0076 (14)
C44	0.052 (2)	0.038 (2)	0.0399 (19)	−0.0180 (17)	−0.0166 (16)	−0.0037 (16)
C45	0.077 (3)	0.047 (2)	0.042 (2)	−0.033 (2)	−0.010 (2)	−0.0018 (18)
C46	0.078 (3)	0.074 (3)	0.043 (2)	−0.054 (3)	−0.005 (2)	−0.014 (2)
C47	0.047 (2)	0.094 (4)	0.043 (2)	−0.040 (2)	−0.0090 (18)	−0.018 (2)
C48	0.0364 (19)	0.061 (2)	0.0383 (19)	−0.0210 (17)	−0.0097 (15)	−0.0118 (17)
C49	0.0312 (17)	0.0326 (18)	0.0395 (18)	−0.0074 (13)	−0.0187 (14)	−0.0033 (15)
C50	0.057 (2)	0.046 (2)	0.0367 (19)	0.0030 (17)	−0.0245 (17)	−0.0105 (17)
C51	0.079 (3)	0.053 (3)	0.045 (2)	0.011 (2)	−0.039 (2)	−0.0075 (19)
C52	0.054 (2)	0.039 (2)	0.067 (3)	−0.0006 (17)	−0.037 (2)	−0.0041 (19)
C53	0.0342 (19)	0.040 (2)	0.055 (2)	0.0007 (15)	−0.0155 (17)	−0.0071 (18)
C54	0.0338 (18)	0.040 (2)	0.0388 (19)	−0.0051 (15)	−0.0129 (15)	−0.0001 (16)

### Geometric parameters (Å, °)

**Table:**

Cu1—O1	2.040 (2)	C22—H22	0.9500
Cu1—P2	2.2181 (9)	C23—C24	1.384 (5)
Cu1—P1	2.2276 (9)	C23—H23	0.9500
Cu1—O2	2.624 (2)	C24—H24	0.9500
S1—O5	1.439 (2)	C25—C26	1.383 (5)
S1—O4	1.445 (2)	C25—C30	1.388 (4)
S1—N3	1.587 (3)	C26—C27	1.389 (5)
S1—C11	1.766 (3)	C26—H26	0.9500
P1—C25	1.816 (3)	C27—C28	1.380 (5)
P1—C31	1.825 (3)	C27—H27	0.9500
P1—C19	1.831 (3)	C28—C29	1.357 (5)
P2—C49	1.812 (3)	C28—H28	0.9500
P2—C37	1.822 (3)	C29—C30	1.379 (5)
P2—C43	1.825 (3)	C29—H29	0.9500
O1—C1	1.292 (4)	C30—H30	0.9500
O2—C1	1.229 (4)	C31—C32	1.378 (5)
O3—C3	1.351 (3)	C31—C36	1.390 (5)
O3—H3	0.8400	C32—C33	1.390 (5)
N1—N2	1.253 (3)	C32—H32	0.9500
N1—C6	1.414 (4)	C33—C34	1.372 (6)
N2—C8	1.430 (4)	C33—H33	0.9500
N3—C14	1.355 (4)	C34—C35	1.373 (6)
N4—C18	1.354 (4)	C34—H34	0.9500
N4—C14	1.359 (4)	C35—C36	1.378 (5)
N4—H1	0.91 (4)	C35—H35	0.9500
C1—C2	1.500 (4)	C36—H36	0.9500
C2—C7	1.384 (4)	C37—C42	1.380 (4)
C2—C3	1.409 (4)	C37—C38	1.389 (4)
C3—C4	1.383 (4)	C38—C39	1.378 (5)
C4—C5	1.374 (4)	C38—H38	0.9500
C4—H4	0.9500	C39—C40	1.373 (6)
C5—C6	1.390 (4)	C39—H39	0.9500
C5—H5	0.9500	C40—C41	1.371 (5)
C6—C7	1.396 (4)	C40—H40	0.9500
C7—H7	0.9500	C41—C42	1.377 (5)
C8—C13	1.383 (4)	C41—H41	0.9500
C8—C9	1.389 (4)	C42—H42	0.9500
C9—C10	1.384 (4)	C43—C48	1.386 (4)
C9—H9	0.9500	C43—C44	1.388 (5)
C10—C11	1.384 (4)	C44—C45	1.387 (5)
C10—H10	0.9500	C44—H44	0.9500
C11—C12	1.386 (4)	C45—C46	1.370 (6)
C12—C13	1.386 (4)	C45—H45	0.9500
C12—H12	0.9500	C46—C47	1.375 (6)
C13—H13	0.9500	C46—H46	0.9500
C14—C15	1.407 (4)	C47—C48	1.396 (5)
C15—C16	1.361 (5)	C47—H47	0.9500
C15—H15	0.9500	C48—H48	0.9500
C16—C17	1.386 (5)	C49—C50	1.389 (4)
C16—H16	0.9500	C49—C54	1.391 (4)
C17—C18	1.356 (5)	C50—C51	1.385 (5)
C17—H17	0.9500	C50—H50	0.9500
C18—H18	0.9500	C51—C52	1.369 (5)
C19—C24	1.385 (4)	C51—H51	0.9500
C19—C20	1.388 (4)	C52—C53	1.362 (5)
C20—C21	1.385 (5)	C52—H52	0.9500
C20—H20	0.9500	C53—C54	1.384 (5)
C21—C22	1.378 (5)	C53—H53	0.9500
C21—H21	0.9500	C54—H54	0.9500
C22—C23	1.374 (5)		
			
O1—Cu1—P2	114.45 (7)	C21—C22—H22	120.1
O1—Cu1—P1	114.67 (7)	C22—C23—C24	120.2 (3)
P2—Cu1—P1	130.83 (4)	C22—C23—H23	119.9
O1—Cu1—O2	55.07 (8)	C24—C23—H23	119.9
P2—Cu1—O2	102.07 (6)	C23—C24—C19	120.4 (3)
P1—Cu1—O2	103.67 (6)	C23—C24—H24	119.8
O5—S1—O4	117.01 (14)	C19—C24—H24	119.8
O5—S1—N3	114.63 (14)	C26—C25—C30	118.1 (3)
O4—S1—N3	104.26 (14)	C26—C25—P1	116.8 (2)
O5—S1—C11	106.82 (14)	C30—C25—P1	125.0 (2)
O4—S1—C11	106.23 (14)	C25—C26—C27	120.8 (3)
N3—S1—C11	107.27 (14)	C25—C26—H26	119.6
C25—P1—C31	103.77 (15)	C27—C26—H26	119.6
C25—P1—C19	104.45 (14)	C28—C27—C26	119.9 (4)
C31—P1—C19	105.45 (14)	C28—C27—H27	120.1
C25—P1—Cu1	116.38 (10)	C26—C27—H27	120.1
C31—P1—Cu1	109.53 (10)	C29—C28—C27	119.7 (4)
C19—P1—Cu1	116.06 (11)	C29—C28—H28	120.2
C49—P2—C37	104.22 (14)	C27—C28—H28	120.2
C49—P2—C43	104.55 (15)	C28—C29—C30	120.9 (3)
C37—P2—C43	104.23 (14)	C28—C29—H29	119.6
C49—P2—Cu1	114.07 (10)	C30—C29—H29	119.6
C37—P2—Cu1	115.74 (10)	C29—C30—C25	120.7 (3)
C43—P2—Cu1	112.85 (10)	C29—C30—H30	119.6
C1—O1—Cu1	102.76 (19)	C25—C30—H30	119.6
C1—O2—Cu1	77.43 (18)	C32—C31—C36	118.4 (3)
C3—O3—H3	109.5	C32—C31—P1	124.7 (3)
N2—N1—C6	115.7 (2)	C36—C31—P1	116.7 (3)
N1—N2—C8	112.0 (2)	C31—C32—C33	120.3 (4)
C14—N3—S1	122.1 (2)	C31—C32—H32	119.8
C18—N4—C14	124.2 (3)	C33—C32—H32	119.8
C18—N4—H1	123 (2)	C34—C33—C32	120.2 (4)
C14—N4—H1	113 (2)	C34—C33—H33	119.9
O2—C1—O1	123.2 (3)	C32—C33—H33	119.9
O2—C1—C2	121.0 (3)	C33—C34—C35	120.2 (4)
O1—C1—C2	115.7 (3)	C33—C34—H34	119.9
C7—C2—C3	119.2 (3)	C35—C34—H34	119.9
C7—C2—C1	119.4 (3)	C34—C35—C36	119.4 (4)
C3—C2—C1	121.3 (3)	C34—C35—H35	120.3
O3—C3—C4	118.1 (3)	C36—C35—H35	120.3
O3—C3—C2	121.6 (3)	C35—C36—C31	121.3 (4)
C4—C3—C2	120.3 (3)	C35—C36—H36	119.3
C5—C4—C3	120.1 (3)	C31—C36—H36	119.3
C5—C4—H4	120.0	C42—C37—C38	118.3 (3)
C3—C4—H4	120.0	C42—C37—P2	123.6 (2)
C4—C5—C6	120.5 (3)	C38—C37—P2	118.1 (3)
C4—C5—H5	119.7	C39—C38—C37	120.1 (4)
C6—C5—H5	119.7	C39—C38—H38	120.0
C5—C6—C7	119.8 (3)	C37—C38—H38	120.0
C5—C6—N1	115.0 (3)	C40—C39—C38	121.0 (4)
C7—C6—N1	125.2 (3)	C40—C39—H39	119.5
C2—C7—C6	120.1 (3)	C38—C39—H39	119.5
C2—C7—H7	119.9	C41—C40—C39	119.3 (4)
C6—C7—H7	119.9	C41—C40—H40	120.3
C13—C8—C9	120.1 (3)	C39—C40—H40	120.3
C13—C8—N2	116.2 (3)	C40—C41—C42	120.2 (4)
C9—C8—N2	123.7 (3)	C40—C41—H41	119.9
C10—C9—C8	119.7 (3)	C42—C41—H41	119.9
C10—C9—H9	120.2	C41—C42—C37	121.2 (3)
C8—C9—H9	120.2	C41—C42—H42	119.4
C9—C10—C11	120.2 (3)	C37—C42—H42	119.4
C9—C10—H10	119.9	C48—C43—C44	119.0 (3)
C11—C10—H10	119.9	C48—C43—P2	123.3 (3)
C10—C11—C12	120.0 (3)	C44—C43—P2	117.5 (2)
C10—C11—S1	120.2 (2)	C45—C44—C43	120.9 (4)
C12—C11—S1	119.8 (2)	C45—C44—H44	119.5
C11—C12—C13	120.0 (3)	C43—C44—H44	119.5
C11—C12—H12	120.0	C46—C45—C44	119.4 (4)
C13—C12—H12	120.0	C46—C45—H45	120.3
C8—C13—C12	119.9 (3)	C44—C45—H45	120.3
C8—C13—H13	120.0	C45—C46—C47	121.0 (4)
C12—C13—H13	120.0	C45—C46—H46	119.5
N3—C14—N4	113.2 (3)	C47—C46—H46	119.5
N3—C14—C15	131.0 (3)	C46—C47—C48	119.8 (4)
N4—C14—C15	115.8 (3)	C46—C47—H47	120.1
C16—C15—C14	120.4 (3)	C48—C47—H47	120.1
C16—C15—H15	119.8	C43—C48—C47	120.0 (4)
C14—C15—H15	119.8	C43—C48—H48	120.0
C15—C16—C17	121.4 (3)	C47—C48—H48	120.0
C15—C16—H16	119.3	C50—C49—C54	118.7 (3)
C17—C16—H16	119.3	C50—C49—P2	118.2 (2)
C18—C17—C16	118.3 (4)	C54—C49—P2	123.1 (2)
C18—C17—H17	120.9	C51—C50—C49	120.0 (3)
C16—C17—H17	120.9	C51—C50—H50	120.0
N4—C18—C17	119.9 (4)	C49—C50—H50	120.0
N4—C18—H18	120.1	C52—C51—C50	120.5 (3)
C17—C18—H18	120.1	C52—C51—H51	119.7
C24—C19—C20	119.2 (3)	C50—C51—H51	119.7
C24—C19—P1	117.5 (2)	C53—C52—C51	120.1 (3)
C20—C19—P1	123.3 (3)	C53—C52—H52	120.0
C21—C20—C19	120.0 (3)	C51—C52—H52	120.0
C21—C20—H20	120.0	C52—C53—C54	120.4 (3)
C19—C20—H20	120.0	C52—C53—H53	119.8
C22—C21—C20	120.4 (3)	C54—C53—H53	119.8
C22—C21—H21	119.8	C53—C54—C49	120.3 (3)
C20—C21—H21	119.8	C53—C54—H54	119.9
C23—C22—C21	119.8 (3)	C49—C54—H54	119.9
C23—C22—H22	120.1		
			
O1—Cu1—P1—C25	−97.32 (13)	C25—P1—C19—C20	−84.2 (3)
P2—Cu1—P1—C25	85.50 (12)	C31—P1—C19—C20	24.9 (3)
O2—Cu1—P1—C25	−154.86 (12)	Cu1—P1—C19—C20	146.3 (2)
O1—Cu1—P1—C31	145.44 (13)	C24—C19—C20—C21	−2.0 (5)
P2—Cu1—P1—C31	−31.74 (12)	P1—C19—C20—C21	177.5 (3)
O2—Cu1—P1—C31	87.90 (12)	C19—C20—C21—C22	1.1 (5)
O1—Cu1—P1—C19	26.23 (14)	C20—C21—C22—C23	0.2 (6)
P2—Cu1—P1—C19	−150.95 (11)	C21—C22—C23—C24	−0.5 (5)
O2—Cu1—P1—C19	−31.31 (13)	C22—C23—C24—C19	−0.4 (5)
O1—Cu1—P2—C49	9.54 (13)	C20—C19—C24—C23	1.6 (5)
P1—Cu1—P2—C49	−173.28 (11)	P1—C19—C24—C23	−177.9 (3)
O2—Cu1—P2—C49	66.45 (12)	C31—P1—C25—C26	93.1 (3)
O1—Cu1—P2—C37	−111.38 (13)	C19—P1—C25—C26	−156.7 (3)
P1—Cu1—P2—C37	65.80 (13)	Cu1—P1—C25—C26	−27.3 (3)
O2—Cu1—P2—C37	−54.47 (13)	C31—P1—C25—C30	−85.2 (3)
O1—Cu1—P2—C43	128.67 (13)	C19—P1—C25—C30	25.1 (3)
P1—Cu1—P2—C43	−54.15 (13)	Cu1—P1—C25—C30	154.4 (3)
O2—Cu1—P2—C43	−174.42 (13)	C30—C25—C26—C27	2.2 (6)
P2—Cu1—O1—C1	94.71 (18)	P1—C25—C26—C27	−176.1 (4)
P1—Cu1—O1—C1	−82.94 (18)	C25—C26—C27—C28	−1.4 (7)
O2—Cu1—O1—C1	6.85 (17)	C26—C27—C28—C29	−0.4 (7)
O1—Cu1—O2—C1	−7.20 (18)	C27—C28—C29—C30	1.3 (6)
P2—Cu1—O2—C1	−118.72 (18)	C28—C29—C30—C25	−0.4 (6)
P1—Cu1—O2—C1	103.55 (18)	C26—C25—C30—C29	−1.4 (5)
C6—N1—N2—C8	−179.0 (2)	P1—C25—C30—C29	176.9 (3)
O5—S1—N3—C14	−35.7 (3)	C25—P1—C31—C32	15.9 (3)
O4—S1—N3—C14	−164.9 (2)	C19—P1—C31—C32	−93.6 (3)
C11—S1—N3—C14	82.7 (3)	Cu1—P1—C31—C32	140.8 (3)
Cu1—O2—C1—O1	11.2 (3)	C25—P1—C31—C36	−159.4 (3)
Cu1—O2—C1—C2	−165.9 (3)	C19—P1—C31—C36	91.1 (3)
Cu1—O1—C1—O2	−14.5 (4)	Cu1—P1—C31—C36	−34.5 (3)
Cu1—O1—C1—C2	162.8 (2)	C36—C31—C32—C33	−0.1 (5)
O2—C1—C2—C7	3.6 (4)	P1—C31—C32—C33	−175.3 (3)
O1—C1—C2—C7	−173.7 (3)	C31—C32—C33—C34	−0.2 (6)
O2—C1—C2—C3	−179.8 (3)	C32—C33—C34—C35	−0.6 (6)
O1—C1—C2—C3	2.8 (4)	C33—C34—C35—C36	1.6 (6)
C7—C2—C3—O3	178.9 (3)	C34—C35—C36—C31	−1.9 (6)
C1—C2—C3—O3	2.3 (4)	C32—C31—C36—C35	1.1 (5)
C7—C2—C3—C4	0.0 (4)	P1—C31—C36—C35	176.7 (3)
C1—C2—C3—C4	−176.6 (3)	C49—P2—C37—C42	99.5 (3)
O3—C3—C4—C5	−178.6 (3)	C43—P2—C37—C42	−9.9 (3)
C2—C3—C4—C5	0.4 (5)	Cu1—P2—C37—C42	−134.4 (3)
C3—C4—C5—C6	−0.4 (5)	C49—P2—C37—C38	−81.2 (3)
C4—C5—C6—C7	0.0 (5)	C43—P2—C37—C38	169.4 (3)
C4—C5—C6—N1	−179.7 (3)	Cu1—P2—C37—C38	44.9 (3)
N2—N1—C6—C5	174.7 (3)	C42—C37—C38—C39	−0.3 (6)
N2—N1—C6—C7	−5.0 (4)	P2—C37—C38—C39	−179.7 (3)
C3—C2—C7—C6	−0.3 (4)	C37—C38—C39—C40	1.0 (7)
C1—C2—C7—C6	176.3 (3)	C38—C39—C40—C41	−0.8 (7)
C5—C6—C7—C2	0.3 (4)	C39—C40—C41—C42	0.0 (7)
N1—C6—C7—C2	180.0 (3)	C40—C41—C42—C37	0.6 (6)
N1—N2—C8—C13	165.2 (3)	C38—C37—C42—C41	−0.4 (5)
N1—N2—C8—C9	−15.2 (4)	P2—C37—C42—C41	178.9 (3)
C13—C8—C9—C10	−3.7 (5)	C49—P2—C43—C48	1.7 (3)
N2—C8—C9—C10	176.7 (3)	C37—P2—C43—C48	110.9 (3)
C8—C9—C10—C11	1.3 (5)	Cu1—P2—C43—C48	−122.8 (2)
C9—C10—C11—C12	1.2 (5)	C49—P2—C43—C44	176.5 (2)
C9—C10—C11—S1	−177.9 (3)	C37—P2—C43—C44	−74.4 (3)
O5—S1—C11—C10	−1.2 (3)	Cu1—P2—C43—C44	51.9 (3)
O4—S1—C11—C10	124.4 (3)	C48—C43—C44—C45	−0.2 (5)
N3—S1—C11—C10	−124.5 (3)	P2—C43—C44—C45	−175.1 (3)
O5—S1—C11—C12	179.7 (3)	C43—C44—C45—C46	0.3 (5)
O4—S1—C11—C12	−54.7 (3)	C44—C45—C46—C47	−0.5 (6)
N3—S1—C11—C12	56.4 (3)	C45—C46—C47—C48	0.6 (6)
C10—C11—C12—C13	−1.4 (5)	C44—C43—C48—C47	0.2 (5)
S1—C11—C12—C13	177.6 (3)	P2—C43—C48—C47	174.9 (2)
C9—C8—C13—C12	3.5 (5)	C46—C47—C48—C43	−0.4 (5)
N2—C8—C13—C12	−176.9 (3)	C37—P2—C49—C50	162.7 (3)
C11—C12—C13—C8	−0.9 (5)	C43—P2—C49—C50	−88.2 (3)
S1—N3—C14—N4	173.9 (2)	Cu1—P2—C49—C50	35.5 (3)
S1—N3—C14—C15	−5.7 (5)	C37—P2—C49—C54	−17.5 (3)
C18—N4—C14—N3	176.8 (3)	C43—P2—C49—C54	91.7 (3)
C18—N4—C14—C15	−3.4 (5)	Cu1—P2—C49—C54	−144.6 (2)
N3—C14—C15—C16	−177.6 (3)	C54—C49—C50—C51	−2.4 (5)
N4—C14—C15—C16	2.8 (5)	P2—C49—C50—C51	177.5 (3)
C14—C15—C16—C17	−0.7 (6)	C49—C50—C51—C52	0.7 (6)
C15—C16—C17—C18	−1.0 (6)	C50—C51—C52—C53	1.6 (6)
C14—N4—C18—C17	1.9 (5)	C51—C52—C53—C54	−2.1 (6)
C16—C17—C18—N4	0.4 (6)	C52—C53—C54—C49	0.3 (5)
C25—P1—C19—C24	95.4 (3)	C50—C49—C54—C53	1.9 (5)
C31—P1—C19—C24	−155.6 (2)	P2—C49—C54—C53	−178.0 (3)
Cu1—P1—C19—C24	−34.2 (3)		

### Hydrogen-bond geometry (Å, °)

**Table:**

*D*—H···*A*	*D*—H	H···*A*	*D*···*A*	*D*—H···*A*
C46—H46···N3^i^	0.95	2.59	3.542 (5)	178
C18—H18···O4^ii^	0.95	2.49	3.042 (4)	117
C5—H5···O5^iii^	0.95	2.49	3.429 (4)	169
N4—H1···O4^ii^	0.91 (4)	2.36 (4)	2.957 (4)	123 (3)
N4—H1···N3^ii^	0.91 (4)	2.20 (4)	3.112 (4)	171 (3)
O3—H3···O1	0.84	1.76	2.510 (3)	147

Symmetry codes: (i) *x*−1, *y*−1, *z*; (ii) −*x*+2, −*y*+2, −*z*+1; (iii) −*x*+2, −*y*+2, −*z*.
